# Biofilm Formation and Resistance to Fungicides in Clinically Relevant Members of the Fungal Genus *Fusarium*

**DOI:** 10.3390/jof4010016

**Published:** 2018-01-23

**Authors:** Hafize Sav, Haleh Rafati, Yasemin Öz, Burcu Dalyan-Cilo, Beyza Ener, Faezeh Mohammadi, Macit Ilkit, Anne D. van Diepeningen, Seyedmojtaba Seyedmousavi

**Affiliations:** 1 Department of Mycology, Kayseri Education and Research Hospital, Kayseri 38010, Turkey; hafize.sav@hotmail.com; 2 Department of Microbiology and Immunology, Center of Excellence for Infection Biology and Antimicrobial Pharmacology, Tehran 1969753491, Iran; halehrafati@gmail.com; 3 Division of Mycology, Department of Microbiology, Faculty of Medicine, University of Osmangazi, Eskişehir 26040, Turkey; dryaseminoz@gmail.com; 4 Division of Mycology, Department of Medical Microbiology, University of Health Sciences Bursa High Specialization Training and Research Hospital, Bursa 16320, Turkey; bdalyan@yahoo.com; 5 Department of Microbiology, Faculty of Medicine, Uludağ University, Bursa 16059, Turkey; bener@uludag.edu.tr; 6 Department of Medical Parasitology and Mycology, School of Medicine, Qazvin University of Medical Sciences, Qazvin 34156-13911, Iran; faezehmohamadi119@yahoo.com; 7 Division of Mycology, Department of Microbiology, Faculty of Medicine, University of Çukurova, Adana 01330, Turkey; macitilkit@gmail.com; 8 Westerdijk Fungal Biodiversity Institute, 3584 CT Utrecht, The Netherlands; anne.vandiepeningen@wur.nl; 9 Department of Medical Microbiology, Center of Expertise in Mycology Radboudumc/CWZ, 6500 HB Nijmegen, The Netherlands; 10 Invasive Fungi Research Center, Mazandaran University of Medical Sciences, Sari 48175-1665, Iran

**Keywords:** biofilms, *Fusarium solani* species complex, *Fusarium petroliphilum*, *Fusarium keratoplasticum*, antifungal resistance

## Abstract

Clinically relevant members of the fungal genus, *Fusarium*, exhibit an extraordinary genetic diversity and cause a wide spectrum of infections in both healthy individuals and immunocompromised patients. Generally, *Fusarium* species are intrinsically resistant to all systemic antifungals. We investigated whether the presence or absence of the ability to produce biofilms across and within *Fusarium* species complexes is linked to higher resistance against antifungals. A collection of 41 *Fusarium* strains, obtained from 38 patients with superficial and systemic infections, and three infected crops, were tested, including 25 species within the *Fusarium fujikuroi* species complex, 14 from the *Fusarium solani* species complex (FSSC), one *Fusarium dimerum* species complex, and one *Fusarium oxysporum* species complex isolate. Of all isolates tested, only seven strains from two species of FSSC, five *F*. *petroliphilum* and two *F*. *keratoplasticum* strains, recovered from blood, nail scrapings, and nasal biopsy samples, could produce biofilms under the tested conditions. In the liquid culture tested, sessile biofilm-forming *Fusarium* strains exhibited elevated minimum inhibitory concentrations (MICs) for amphotericin B, voriconazole, and posaconazole, compared to their planktonic counterparts, indicating that the ability to form biofilm may significantly increase resistance. Collectively, this suggests that once a surface adherent biofilm has been established, therapies designed to kill planktonic cells of *Fusarium* are ineffective.

## 1. Introduction

Despite being well known as plant pathogens, *Fusarium* species (order *Hypocraeles*) cause a broad spectrum of superficial infections, such as keratitis and onychomycosis, as well as locally invasive and disseminated fusarioses in human and animals [1,2]. The genus *Fusarium* also contains species which may spoil crops by the production of persistent mycotoxins that affectconsumers’ health [1].

At present, the genus *Fusarium* consists of more than 200 species, divided in 22 species complexes, differing by morphology, host association, and molecular characteristics [3,4]. Among them, the *Fusarium solani* species complex (FSSC) and *Fusarium oxysporum* species complex (FOSC) are responsible for approximately 60% and 20% of human fusariosis, respectively [5,6,7]. Importantly, clinically relevant members of the genus *Fusarium* display high levels of resistance to systemic azoles, echinocandins, and polyenes [8,9,10,11,12]. The antifungal susceptibility within each species complex also varies from one species to another, which poses a major challenge in the management of patients with *Fusarium* infections [6,13].

In human pathogenic fungi, such as *Candida* and *Aspergillus*, biofilm formation increases fungal resistance to antifungal compounds [14,15,16,17], while it also plays a role in the colonization of specific surfaces [18,19]. The biofilm-forming ability of *Fusarium* strains, and its link with reduced antifungal susceptibility, has been reported in keratitis patients [20,21,22,23]. Previous studies also reported the possibility of biofilm-formation on contact lenses in outbreaks of keratitis caused by *Fusarium* species [21,22,24]. We therefore investigated whether various levels of biofilm formation or absence of this feature, across and within *Fusarium* species complexes, are linked to higher resistance against systemic antifungals. 

## 2. Materials and Methods

### 2.1. Fungal Strains

A collection of 38 clinical *Fusarium* strains obtained from 38 patients with superficial and systemic infections, and 3 isolates from *Fusarium* infections in crops, was used. Table 1 describes the reference numbers of the isolates, the species complexes they reside in, sources, geographic origins, and in the case of clinical infections, the underlying disease, for all of the *Fusarium* strains. All strains were obtained from the reference collection of the CBS-KNAW Fungal Biodiversity Center (housed at Westerdijk Fungal Biodiversity Institute, Utrecht, The Netherlands) and handled under biosafety laboratory regulations. Identity of the organisms was confirmed by sequencing of the internal transcribed spacer regions of rDNA, translation elongation factor 1α (*TEF1*α) and the RNA polymerase II gene (*RPB2*), as described previously [6]. Prior to testing, all isolates were subcultured on Sabouraud glucose agar (Merck, Darmstadt, Germany), at 25 °C for 3–5 days.

### 2.2. Antifungal Susceptibility Testing of Planktonic Cells

The planktonic cells of each *Fusarium* isolate were tested for in vitro susceptibility to amphotericin B (AMB; Bristol–Myers Squibb, Woerden, The Netherlands), anidulafungin (AND; Pfizer Central Research, Sandwich, Tadworth, Surrey, UK), caspofungin (CAS; Merck Sharp & Dohme BV, Haarlem, The Netherlands), fluconazole (FLC; Pfizer Central Research Sandwich, Tadworth, Surrey, UK), flucytosine (5-FC, Sigma–Aldrich, St. Louis, MO, USA), itraconazole (ITC: Janssen Research Foundation, Beerse, Belgium), posaconazole (POS: Merck, Whitehouse Station, NJ, USA), and voriconazole (VOR: Pfizer Central Research, Sandwich, Tadworth, Surrey, UK), by the broth microdilution method, according to the Clinical and Laboratory Standards Institute (CLSI) methodology [25]. Final concentrations of the following antifungal agents ranged from 0.016 to 16 µg/mL: amphotericin B, anidulafungin, caspofungin, itraconazole, posaconazole, and voriconazole. Fluconazole and flucytosine, were assessed over a two-fold concentration range, from 0.064 to 64 µg/mL.

The minimum inhibitory concentrations (MICs) of amphotericin B, flucytosine, fluconazole, itraconazole, voriconazole, and posaconazole were determined visually; an inverted mirror was used for comparing the growth in wells containing the drugs with that in the drug-free control well. The minimum effective concentrations (MECs) of anidulafungin and caspofungin were read using a plate microscope (Olympus SZX9; Olympus Nederland, Zoeterwoude, The Netherlands), at 25× to 50× magnification. *Paecilomyces variotii* (ATCC 22319), *Candida parapsilosis* (ATCC 22019), and *C. krusei* (ATCC 6258) were used for quality controls in all experiments. All experiments on each strain were performed using three independent replicates on different days. The geometric means (GMs) MICs and MECs of three independent replicates were determined for each species and drug, after 48 h of incubation. If no growth was observed, or the growth was not adequate, the incubation was extended to 72 h.

### 2.3. In Vitro Biofilm Formation Assay

The ability of the *Fusarium* strains to form biofilms was tested using the Crystal violet staining method in three independent replicates, as described previously [26]. Briefly, conidial suspension of each strain was adjusted to a final concentration of 1 × 10^6^ conidia/mL in phosphate-buffered saline (PBS). One hundred µL of this suspension was placed in a tube containing 2 mL of brain-heart infusion broth (BHIB) with glucose (0.25%). The tubes were incubated at 37 °C for 24 h, and the suspensions were diluted in a ratio of 1:20 in freshly prepared BHIB with glucose. A 200-μL aliquot of this suspension was added to each well of a flat-bottom 96-well polystyrene microtiter plate (Corning Inc., Corning, NY, USA). After incubation for 24 h at 37 °C, the microplate was rinsed three times with PBS, and then inverted to drain, and 200-μL of 1% crystal violet was added to each well. After incubation for 15 min at room temperature, the microplate was again rinsed three times with PBS. Next, 200-μL of an ethanol:acetone mixture (80:20 *w*/*v*) was added to each well. The plates were read at 450 nm using a plate reader (Biotek EL × 808, Winooski, VT, USA). The percent transmittance (%T) value of each test sample was subtracted from the %T value of the reagent blank to obtain a measure of the relative amount of light blocked by the sample (%T_bloc_). The biofilm production of each isolate was considered negative (%T_bloc_, <5), + (%T_bloc_, 5–20), ++ (%T_bloc_, 20–50), or +++ (%T_bloc_, >50). The biofilm activity of *C. albicans* ATCC 92228 (%T_bloc_, 5–20) was considered the positive quality control.

### 2.4. Antifungals Susceptibility of Pre-Formed Biofilms

To analyze the effects of antifungals on pre-formed biofilms, *Fusarium* biofilms were first established on the surface of 96-well, flat-bottomed microtiter plates, as described previously [27]. Briefly, the planktonic cell suspensions of 1 × 10^6^ cells/mL in PBS, containing 0.025% (*v*/*v*) Tween-20, were prepared, and 200-μL was added to select wells and the suspensions were incubated at 37 °C for 24 h. Then, non-adherent cells were removed by washing with PBS, and a 200-μL RPMI 1640 medium, containing various antifungal concentrations, was added to the selected wells and incubated at 37 °C for an additional 24 h. Negative-control wells received 200-μL RPMI 1640 alone. The effects of antifungals on the pre-formed biofilms were then estimated using a semi-quantitative viability based XTT (2,3-bis(2-methoxy-4-nitro-5-sulfophenyl)-5-[(phenylamino) carbonyl]-2*H*-tetrazolium hydroxide) reduction assay, within 2 h of incubation, at 35 °C to 37 °C, as described previously [27,28]. Briefly, XTT (Sigma–Aldrich, St Louis, MO, USA) was prepared as a saturated solution (0.5 g/liter) in PBS. The solution was filter sterilized through a 0.22 µm pore-size filter, aliquoted, and stored at −70 °C. Prior to use, an aliquot of stock XTT was thawed, and 10 mM menadione (Sigma–Aldrich, St Louis, MO, USA), prepared in acetone, was added to the XTT, to make a final concentration of 1 µM. Subsequently, 100 µL of the above-mentioned XTT-menadione solution was added to each pre-washed biofilm, and to the control wells, to measure background XTT levels. The plates were further incubated at 37 °C for 2 h, in order to allow conversion of XTT to its formazan derivative. XTT conversion, as a direct correlation of the metabolic activity of the biofilm, was then measured as optical density (OD), with a microtitration plate spectrophotometric reader (Biotek EL × 808, USA) at 450 nm/630 nm. For each well, XTT conversion was calculated after subtraction of the background OD, which was the OD of a simultaneously incubated well with 100 µL of XTT-menadione solution, but no biofilm. Percentages of fungal growth were calculated for each well by dividing the XTT conversion of each well by the XTT conversion of the drug-free growth control well.

### 2.5. Data Analysis

Data were analyzed using GraphPad Prism, version 5.0, for Windows (GraphPad Software, San Diego, CA, USA). MIC/MEC distributions between the groups were compared using Student’s *t* test and the Mann–Whitney–Wilcoxon test; differences were considered statistically significant at *p* values of ≤0.05 (two-tailed).

## 3. Results

### 3.1. Antifungal Susceptibility Profile of Planktonic Cells

As shown in Table 1, amphotericin B had the highest in vitro activity against the planktonic form of all *Fusarium* species tested, with the MIC ranging from 0.125 to 8 µg/mL. Both voriconazole and posaconazole showed interspecies variability, across and within *Fusarium* species complexes, with the MIC ranging from 1 to 16 µg/mL and 0.125 to >16 µg/mL, respectively. However, all the species indiscriminately showed high MIC/MEC values for flucytosine, fluconazole, itraconazole, anidulafungin, and caspofungin. The MICs/MECs were identical between replicates.

### 3.2. Biofilm Formation

Of all isolates tested, only seven strains from FSSC, including five *F*. *petroliphilum* strains and two *F*. *keratoplasticum* strains, scored a %T_bloc_ 20–50, displaying the capacity to form biofilms. The *F. petroliphilum* strains were identified in blood (*n* = 4), and nasal biopsy (*n* = 1) samples of patients with underlying acute lymphoblastic leukemia and myelodysplastic syndrome, respectively, while the *F. keratoplasticum* strains (*n* = 2) were recovered from nail scrapings of onychomycosis patients. The ability to produce biofilms was not detected in the remaining 34 strains.

### 3.3. Sessile Susceptibilities of Fusarium Strains

Amphotericin B showed the lowest MIC values against planktonic cells of all biofilm-positive species, with the MIC ranging from 0.25 to 2 µg/mL, but did not significantly differ from most other species (*p* > 0.05). Biofilm-forming strains of the two species showed higher MIC to azoles and echinocandins, whereas non-biofilm forming species had more variability in their susceptibility to these compounds. Of note, intraspecies variation exhibited within *F. keratoplasticum* and *F. proliferatum* species, with the MIC ranging 8 to 16 µg/mL, and 0.125 to >16 µg/mL for voriconazole and posaconazole, respectively.

The sessile MICs, determined against *Fusarium* biofilms formed in microtiter plates, were significantly higher than planktonic MICs (*p* ≤ 0.05) for amphotericin B, voriconazole and posaconazole, ranging from 2 to 8 μg/mL, >16 μg/mL, and 0.5 to >16 μg/mL, respectively, while no significant differences were found for echinocandins (Table 2).

## 4. Discussion

Our study showed that the seven tested isolates of two species, *F*. *petroliphilum* and *F*. *keratoplasticum*, both from the FSSC, could produce biofilms. These strains were recovered from blood, nails, and nasal biopsies of superficial and systemic fusariosis, but so were many of the non-biofilm producing strains, indicating that the biofilm formation trait is not the main contributing factor that causes these infections in the genus, *Fusarium.* The biofilm formation has been shown a major virulence attribute of pathogenicity in medically important fungi, such as *Candidia*, *Aspergillus*, and *Pseudallescheria/Scedosporium* species [17,19,29,30,31]. In *Fusarium*, the ability to form biofilms was suggested as a pathogenicity determinant in an outbreak of fusarial keratitis, irrespective of the thickness of these biofilms [21].

The planktonic forms of biofilm-forming isolates all showed high resistance to tested azoles and echinocandins, whereas non-biofilm producers showed more variation and some of these were less resistant to these compounds. In addition, in the liquid culture tested, sessile *Fusarium* biofilms exhibited elevated MICs, compared to their planktonic counterparts, for amphotericin B, voriconazole, and posaconazole, indicating that the ability to form a biofilm may significantly (*p* ≤ 0.05) increase resistance, as shown in Table 2. This suggests that once a surface adherent biofilm has been established, therapies designed to kill planktonic cells of *Fusarium* are ineffective.

Similarly, Zhang et al. [20] reported that *Fusarium* species producing mature biofilms were intrinsically resistant to azole antifungal compounds. In another study, Imamura et al. [22] also observed that *Fusarium* biofilms in contact lenses may reduce susceptibility to lens care solutions in a time-dependent manner, suggesting that this extracellular matrix prevents antifungal penetration or that the biofilm increases the expression of a drug efflux pump system [14,15,32]. In contrast, however, Mukherjee et al. [21] reported that biofilms had no apparent effect on the natamycin susceptibility of FSSC and FOSC; voriconazole was active against biofilms formed by FSSC, and amphotericin B was active against FOSC.

In our study, we only tested one *F. oxysporum* strain, which did not form detectable biofilms. However, Mukherjee et al. showed that members of FOSC were able to produce (lower) levels of biofilms [21]. *F. oxysporum* is reported from localized and disseminated life-threatening opportunistic infections in immunocompetent and severely neutropenic patients. Studies have also shown that clinically important lineages of *F. oxysporum* are linked with water systems in hospitals [33], supporting the possibility of nosocomial *F. oxysporum* infections. In addition to *F. oxysporum*, none of the tested *F. solani* strains originating from superficial and systemic infections had the ability to form biofilms, which is in contrast with previous reports suggesting that the ability of *F. solani* to form biofilms on contact lenses may have had a role in the keratitis outbreak [20,21,22,24].

Importantly, *F*. *petroliphilum* and *F. keratoplasticum*, the two biofilm-forming species in our study, were abundantly found in sinks and drains—man-made environments typically inhabited by biofilm-forming microorganisms [34]. Collectively, this suggests that the biofilm formation may also be a trait that also enables a species to establish itself in common human environments where people, including immunocompromised patients vulnerable to infection, may encounter them.

*Fusarium* strains generally show high intrinsic levels of resistance to the tested antifungal drugs. The underlying mechanisms leading to antifungal resistance in *Fusarium* are not yet understood, and a complex of involved regulatory proteins, enzymes, and transporter genes is suggested [35]. The observed mechanism of increased resistance in *Fusarium* species includes specific transcription regulators, such as CCG-8 [36], up-regulated ABC-transporters [35,37], and in the case of azole resistance, the presence of three lanosterol 14 alpha-demethylase paralogues (*CYP*51A, B, and C) [38]. Some of these mechanisms may also be involved in the antifungal resistance in *Fusarium* biofilms. Several studies have demonstrated that in *Candida* species, biofilm formation leads to dramatically increased levels of resistance to the most commonly used antifungal agents [39], and that the reason is multifactorial (mechanical, biochemical, and genetical factors); one mechanism of the increased resistance proves to be the up-regulation of efflux pumps and other resistance genes [15], as well as increased metabolically activity [18], during the development of biofilms. The three-dimensional architecture of the biofilm with increased cell densities and the formed extracellular “exopolymeric substance” (EPS) matrix have been found to be important factors [40]. Furthermore, nutrient limitation in biofilms may influence growth rates, and phenotypically altered “persister” cells are typically formed in the biofilms [41]. Also, in *Aspergillus fumigatus*, biofilm formation has been shown to increase antifungal drug resistance [16], with multifactorial principles, including the formation of extra cellular DNA (eDNA), to stabilize the EPS matrix [42].

## 5. Conclusions

*Fusarium* species are emerging in human infections. Biofilm formation is a relatively common feature in fungal etiological agents that renders biofilm-producing *Fusaria* even more refractory to treatment, while non-biofilm producing strains already possess a high level of innate resistance to most antifungal drugs available. The fact that biofilm production also allows the fungi to establish themselves well in human-made environments, like sinks and bathrooms, where they can act as reservoirs for nosocomial infections, makes them even a more serious threat to humans. Further studies, however, are warranted, to explore this association in greater detail, and to determine the mechanisms of virulence and antifungal resistance in biofilm-producing *Fusarium* species.

## Figures and Tables

**Table 1:** Clinical origins, characteristics, the ability to form biofilms and in vitro MICs/MECs, obtained by Clinical and Laboratory Standards Institute (CLSI) susceptibility testing of eight antifungal agents against planktonic cells of the collection of 41 *Fusarium* isolates tested in this study.

No		CBS No	Species	Source	Country	Underlying Disease	AMB	5-FC	FLC	ITR	VRC	POS	AND	CAS	Biofilm Formation
							Planktonic MIC/MEC (μg/mL)								
1	*Fusarium dimerum* species complex	139002	*F. dimerum*	Skin biopsy	Turkey	Paraplegia	0.5	>64	>64	>64	4	>16	>16	>16	−
2	*Fusarium fujikuroi* species complex	139195	*F. andiyazi*	Blood	Turkey	Acute myeloid leukemia	8	>64	16	8	2	1	>16	8	−
3		138998	*F. proliferatum*	Blood	Turkey	Acute lymphoblastic leukemia	0.5	>64	>64	>64	4	>16	>16	>16	−
4		138924	*F. proliferatum*	Nasal biopsy	Turkey	Acute myeloid leukemia	0.125	>64	>64	>64	4	>16	>16	>16	−
5		138925	*F. proliferatum*	Skin biopsy	Turkey	Chronic renal failure	0.125	>64	>64	>64	4	>16	>16	>16	−
6		139000	*F. proliferatum*	BAL	Turkey	Aplastic anemia	0.25	>64	>64	>64	1	0.125	>16	>16	−
7		139001	*F. proliferatum*	Skin biopsy	Turkey	Acute lymphoblastic leukemia	1	>64	>64	>64	4	1	>16	>16	−
8		139003	*F. proliferatum*	Blood	Turkey	Acute lymphoblastic leukemia	1	>64	>64	>64	4	>16	>16	>16	−
9		139004	*F. proliferatum*	Sputum	Turkey	Lung cancer	0.5	>64	>64	>64	4	>16	>16	>16	−
10		138929	*F. proliferatum*	Cornea scraping	Turkey	Keratitis	1	>64	>64	>64	4	>16	>16	>16	−
11		138930	*F. proliferatum*	Nasal biopsy	Turkey	Aplastic anemia	1	>64	>64	>64	4	1	>16	>16	−
12		138928	*F. proliferatum*	Blood	Turkey	Malign melanoma	1	>64	>64	>64	2	0.5	>16	>16	−
13		139198	*F. proliferatum*	Nasal biopsy	Turkey	Acute myeloid leukemia	1	>64	>64	>64	8	>16	>16	>16	−
14		138933	*F. proliferatum*	Nasal biopsy	Turkey	Acute lymphoblastic leukemia	1	>64	>64	>64	4	0.5	>16	>16	−
15		138930	*F. proliferatum*	Nasal biopsy	Turkey	Aplastic anemia	1	>64	>64	>64	4	1	>16	>16	−
16		480.77	*F. proliferatum*	Banana, bud rot	the Netherlands	-	1	>64	>64	>16	2	1	>16	>16	−
17		133030	*F. proliferatum*	Nail scraping	Iran	Onychomycosis	0.5	>64	>64	>16	8	>16	>16	>16	−
18		131391	*F. proliferatum*	Wheat root	Australia	-	0.5	>64	>64	>16	8	>16	>16	>16	−
19		130179	*F. proliferatum*	Blood	USA	-	1	>64	>64	>64	4	2	>16	>16	−
20		139015	*F. verticillioides*	Blood	Turkey	Acute myeloid leukemia	2	>64	>64	>64	1	0.25	>16	>16	−
21		139018	*F. verticillioides*	Blood	Turkey	T-cell lymphoma	4	>64	>64	>64	1	0.125	>16	>16	−
22		139202	*F. verticillioides*	Blood	Turkey	Acute lymphoblastic leukemia	4	>64	>64	>64	1	0.25	>16	>16	−
23		579.78	*F. verticillioides*	Leg ulcer	USA	Left leg ulcer	2	>64	>64	16	1	0.25	>16	>16	−
24		123670	*F. verticillioides*	Maize	USA	-	2	>64	64	16	2	1	>16	>16	−
25		115135	*F. verticillioides*	Blood	Sweden	-	2	>64	>64	>16	2	0.5	>16	>16	−
26		108922	*F. verticillioides*	Urine	Germany	-	2	>64	>64	>16	1	0.25	>16	>16	−
27	*Fusarium oxysporum* species complex	138926	*F. oxysporum*	Sputum	Turkey	Hepatic cirrhosis	0.5	>64	>64	>64	2	>16	>16	>16	−
28	*Fusarium solani* species complex	139005	*F. keratoplasticum*	Nail scraping	Turkey	Onychomycosis	2	>64	>64	>64	8	0.125	>16	>16	+
29		139017	*F. keratoplasticum*	Nail scraping	Turkey	Onychomycosis	2	>64	>64	>64	8	>16	>16	>16	+
30		139006	*F. petroliphilum*	Blood	Turkey	Acute myeloid leukemia	0.25	>64	>64	>64	16	>16	>16	>16	+
31		138932	*F. petroliphilum*	Nasal biopsy	Turkey	Myelodysplastic syndrome	0.5	>64	>64	>64	8	>16	>16	>16	+
32		139011	*F. petroliphilum*	Blood	Turkey	Acute lymphoblastic leukemia	1	>64	>64	>64	8	>16	>16	>16	+
33		139324	*F. petroliphilum*	Blood	Turkey	Acute lymphoblastic leukemia	0.5	>64	>64	>64	8	>16	>16	>16	+
34		139013	*F. petroliphilum*	Blood	Turkey	Acute lymphoblastic leukemia	1	>64	>64	>64	8	>16	>16	>16	+
35		139016	*F. petroliphilum*	Nail scraping	Turkey	Onychomycosis	1	>64	>64	>64	8	>16	>16	>16	−
36		139205	*F. solani*	Sputum	Turkey	Larynx cancer	1	>64	>64	>64	4	>16	>16	>16	−
37		139007	*F. solani*	Skin scraping	Turkey	Diabetes	1	>64	>64	>64	8	>16	>16	>16	−
38		139008	*F. solani*	Nasal biopsy	Turkey	Acute myeloid leukemia	1	>64	>64	>64	2	>16	>16	>16	−
39		139012	*F. solani*	Cornea scraping	Turkey	Keratitis	0.25	>64	>64	>64	2	>16	>16	>16	−
40		139200	*F. solani*	Cornea scraping	Turkey	Keratitis	1	>64	>64	>64	8	>16	>16	>16	−
41		139197	*F. solani*	Skin biopsy	Turkey	Acute myeloid leukemia	2	>64	>64	>64	2	>16	>16	>16	−

MIC: Minimum Inhibitory Concentration, MEC: Minimum effective concentrations, AMB: amphotericin B, 5-FC: flucytosine, FLC: fluconazole, ITC: itraconazole, VRC: voriconazole, POS: posaconazole, AFG: anidulafungin, CAS: caspofungin, BAL: Bronchoalveolar lavage. The positive signs (+) indicate the ability to produce biofilm, and the negative signs (−) show lack of biofilm formation. The isolates with the ability to produce biofilms are highlighted in gray color.

**Table 2:** Comparison of planktonic and sessile susceptibilities of biofilm-forming *Fusarium* isolates.

CBS No	Species	Biofilm Formation	AMB		ITC		VRC		POS		AND		CAS	
			P_MIC_	S_MIC_	P_MIC_	S_MIC_	P_MIC_	S_MIC_	P_MIC_	S_MIC_	P_MIC_	S_MIC_	P_MIC_	S_MIC_
			(μg/mL)											
139005	*F. keratoplasticum*	+	2	2	>16	>16	8	>16	0.125	0.5	>16	>16	>16	>16
139017	*F. keratoplasticum*	+	2	8	>16	>16	8	>16	>16	>16	>16	>16	>16	>16
139006	*F. petroliphilum*	+	0.25	2	>16	>16	16	>16	>16	>16	>16	>16	>16	>16
138932	*F. petroliphilum*	+	0.5	4	>16	>16	8	>16	>16	>16	>16	>16	>16	>16
139011	*F. petroliphilum*	+	1	2	>16	>16	8	>16	>16	>16	>16	>16	>16	>16
139324	*F. petroliphilum*	+	0.5	4	>16	>16	8	>16	>16	>16	>16	>16	>16	>16
139013	*F. petroliphilum*	+	1	2	>16	>16	8	>16	>16	>16	>16	>16	>16	>16

P: planktonic, S: sessile. The positive signs (+) indicate the ability to produce biofilm.
